# A prospective and randomized comparison of rigid ureteroscopic to flexible cystoscopic retrieval of ureteral stents

**DOI:** 10.1186/s12894-017-0220-8

**Published:** 2017-04-21

**Authors:** Dehui Lai, Meiling Chen, Shifang Zha, Shawpong Wan

**Affiliations:** 1 Urology Department, Fifth Affiliated Hospital, Guangzhou Medical University, 621 Gangwan Road, Huangpu District, Guangzhou, 510700 China; 2 Urology, Citic Huizhou Hospital, Huizhou, Guangdong China; 3 Urology, First People’s Hospital of Xiaoshan, Hangzhou, Zhejiang China

**Keywords:** Ureteral stents, Stent retrieval, Cost-effectiveness

## Abstract

**Background:**

Flexible cystoscopy has become an accepted alternative for stent retrieval. However, it is associated with higher cost. Some reports have described experiences of using rigid ureteroscope to retrieve ureteral stents. We compared rigid ureteroscopic to flexible cystoscopic retrieval of ureteral stents in a prospective and randomized clinical trial.

**Methods:**

Three hundred patients treated with ureteral stents between July 2012 and July 2013 were accrued in this study. These patients were divided into two groups using the random number table method. Group A, with 162 patients, had stents removed with a flexible cystoscope and Group B, with 138 patients, had stents removed with a rigid ureteroscope. All procedures were performed under topical anesthesia by the same urologist. Patients in each group were compared in terms of preoperative, perioperative, and postoperative data. Postoperative data were collected using telephone interview on the postoperative day two. The postoperative questionnaire used included three items: hematuria, irritable bladder symptoms, and pain scores.

**Results:**

All the stents were retrieved successfully. No statistical differences were noted between the two groups in terms of gender, age, laterality and duration of the stents, operative time, postoperative hematuria, irritable bladder symptoms, and pain scores. The per-use cost of instrument was much higher for the flexible cystoscopic group, RMB 723.1 versus 214.3 (USD 107.9 versus 28.2), *P* < 0.05.

**Conclusion:**

Ureteral stent retrieval using rigid ureteroscope under topical anesthesia is as safe and effective as flexible cystoscope but with a much lower cost to patients.

**Trial registration:**

This study was registered with Chinese Clinical Trial Registry on March 27, 2017 (retrospective registration) with a trial registration number of ChiCTR-IOR-17010986.

## Background

Ureteral stents are frequently used in minimally invasive procedures for the upper urinary tract diseases. It may alleviate temporary postoperative obstruction in the ureter from trauma and swelling [1, 2]. It can also be used as a means of passive preoperative ureteral dilation. Conventionally, the indwelling ureteral stents present for a longer period are retrieved using a rigid cystoscope and grasping forceps in adults under topical anesthesia as outpatient. The procedure can be painful and may cause urethral injury in men. Recently, flexible cystoscopy has become an accepted alternative for stent retrieval. However, flexible cystoscope is associated with higher cost and may not be readily available in developing countries [3, 4]. Some reports have described experiences of using rigid ureteroscope to retrieve ureteral stents, especially in the occasional situations such as migrated or retained stents [4–6]. There has been no study published comparing the outcome between using a flexible cystoscope and a rigid ureteroscope for the ureteral stent removal. In this prospective and randomized clinical trial, we intend to investigate this issue.

## Methods

### Patient cohorts

This prospective and randomized trial was approved by the Ethics Committee of the Fifth Affiliated Hospital of the Guangzhou Medical University. From July 2012 to July 2013, 300 adult patients with unilateral 6 Fr. double-J ureteral stents were accrued for the study. Patients with residual stones, chronic renal failure, diabetes, solitary kidney, history of sepsis, febrile infection, or migrated stents were excluded. Written informed consent was obtained for all participants. Using the random number table, patients were separated into two groups based on the method of stent retrieval. Group A of 162 patients had stents removed using a 16 French Karl Storz flexible cystoscope. Group B of 138 patients had stents removed using a 8.0/9.8 French Richard Wolf ureteroscope. All patients underwent urinalysis and KUB prior to the procedure.

### Procedures and Data collection

All the stents were removed by a single urologist to minimize the variables. First, 2% lidocaine gel was instilled into the urethra and held for 5 min to implement topical anesthesia. Next either a flexible cystoscope or a rigid ureteroscope was introduced into the urethra under direct vision and advanced to the bladder per group assignment. The ureteral stents were removed using either flexible foreign body forceps for the flexible cystoscopic method or the four Fr. rigid grasping forceps for the rigid ureteroscopic method.

The clinical data assessed includes the duration of the stent placement, laterality of the stent, reason for the stent placement, operative time, perioperative and postoperative pain, postoperative hematuria, and irritable bladder symptoms. Operative time was calculated from the insertion of the endoscope to the completion of the stent removal. A visual analogue pain scale (VAS) was used to assess the intensity of the pain. The perioperative and postoperative pain scales were evaluated immediately after the procedure and 48 h after the procedure by telephone. Postoperative macroscopic hematuria lasting more than 1 day after the procedure were recorded. Irritable bladder symptoms included four items: pain in the bladder, dysuria, urinary frequency and urgency. These data were acquired just before the procedure and at 48 h follow up after the procedure by telephone.

The calculated cost of the instruments included the cost of endoscopes, grasping forceps, and maintenance. Per-use cost of the instruments for both groups was appraised and compared.

### Statistical analyses

Statistical analysis was performed using the SPSS 17.0® for Windows®. Continuous variables were compared using the Student-t and the Wilcoxon tests. Univariable analysis was conducted using the Pearson *χ* ^2^ statistics or Fisher’s exact test for the categorical data. *P* values <0.05 were considered statistically significant.

## Results

All stents were successfully removed under topical anesthesia in both groups. There were no statistical differences noted between the two groups in terms of age, gender, laterality of the stent, and the reasons for the stent placement (Table 1).

**Table 1:** Demographic, characteristics of patient, stent laterality, and reason for stent placement

	Flexible cystoscope group (*N* = 162)	Ureteroscope group (*N* = 138)	*P*
Gender, no.			
Male/Female	77/85	74/64	0.293
Mean age ± SD, (range in years)	40.1 ± 10.3, (20–79)	39.6 ± 11.1, (19–68)	0.79
Stent laterality, Left/Right	90/72	73/65	0.645
Causes of stent placement			
MPCNL	97	84	0.996
Ureteroscopic lithotripsy	41	37	
Shock wave lithotripsy	7	5	
Hydronephrosis			
Ureteral stricture	11	8	
Pregnancy	2	1	
Open surgery	4	3	

Duration of the stent, operative time, perioperative and postoperative pain scores, and data for postoperative macroscopic hematuria and irritable bladder symptoms are shown by gender (Tables 2 and 3). The mean operative time was shorter for the ureteroscopic group in both sexes. There was no statistically significant difference between the two groups in both perioperative and postoperative pain scores. When compared to the female patients, the mean perioperative pain score was higher for men in both groups.

**Table 2:** Days from stent placement to removal, perioperative and postoperative characteristics of male patients

	Flexible cystoscope group (*N* = 77)	Ureteroscope group (*N* = 74)	*P*
Variable			
Duration of stent placement mean ± SD, range in days	28.6 ± 9.4,14–32	29.9 ± 9.5,14–36	0.652
Operative time, mean ± SD, range in minutes	3.4 ± 0.8,2.8–4.2	2.7 ± 0.9,2.4–3.5	0.203
VAS score for perioperative pain, mean ± SD, range	3.1 ± 1.8,3–6	4.3 ± 0.9,3–8	0.103
VAS score for postoperative pain, mean ± SD, range	2.4 ± 1.1,1–4	3.1 ± 1.2,1–5	0.324
Patients lost at follow up, n (%)	8 (10.4)	6 (8.1)	0.629
Postoperative macroscopic hematuria, n (%)			
More than 1 day	14 (20.3)	18 (26.4)	0.395
Postoperative irritable bladder symptoms, n (%)	19 (29.2)	23 (33.8)	0.425

**Table 3:** Duration of the stents, perioperative and postoperative characteristics of female patients

	Flexible cystoscope group (*N* = 85)	Ureteroscope group (*N* = 64)	*P*
Variable			
Duration of stents, mean ± SD, range, days	27.6 ± 6.5,16–29	28.8 ± 10.5,14–31	0.703
Operative time, mean ± SD, range, minutes	3.0 ± 0.6,2.2–3.5	2.3 ± 0.6,1.5–2.7	0.062
VAS score of perioperative pain, mean ± SD, range	2.5 ± 1.2, 2–6	3.1 ± 1.9, 3–8	0.234
VAS score of postoperative pain, mean ± SD, range	2.3 ± 1.2, 1–4	2.8 ± 1.4, 1–4	0.325
Patients lost at follow up, n (%)	6 (7.1)	7 (10.9)	0.406
Postoperative macroscopic hematuria, n (%)			
More than 1 day	5 (6.3)	5 (8.8)	0.742
Postoperative irritable bladder symptoms, n (%)	6 (7.6)	6 (10.5)	0.382

All patients were discharged 20–30 min after the procedure. No one required analgesics, antibiotics, or hospitalization. Follow-up data was available in 91.4% of the cohorts in Group A and 90.5% in Group B. Four male patients (20.3%) in Group A and 18 (26.4%) in the Group B had postoperative macroscopic hematuria for more than 1 day, *p* = 0.395; whereas only five female patients (6.3%) in Group A and five (8.8%) in group B experienced macroscopic hematuria for more than 1 day, *p* = 0.742. These patients were treated with 5 mg adrenosin once a day and the hematuria ceased in 3 to 5 days. The rates of postoperative irritable bladder symptoms were higher in the ureteroscopic group, especially for men, but there was no statistically significant difference between the two groups. No one required hospitalization due to the postoperative complications. Two forceps and two flexible cystoscopes were damaged during the procedure in Group A. The deflection lever on one of the scopes was severed and the damage on the other was the outer rubber sheath. No ureteroscope and only one pair of grasping forceps was damaged in Group B. The per-use cost for the instrument was much higher in Group A than Group B, RMB 723.1 (USD 107.9) versus RMB 214.3 (USD 28.2) respectively.

## Discussion

Since first introduced in 1967, ureteral stents have been widely used in the urological surgery [5, 7]. As a foreign body, it is generally removed in 3 days to 4 weeks after its insertion. The conventional method for the retrieval is using the widely available rigid cystoscope and grasping forceps. Various non-endoscopic techniques for the stent retrieval have also described. A tethered nylon string attached to the end of the stent is frequently used for stents intended to be used for a short duration. Magnets, wire loops, and crochet hook–like retrievers have also been tried but not widely used [8–13]. With the introduction of flexible cystoscopes, the flexible cystoscopic stent removal has become a preferred method in the more affluent countries. However, flexible cystoscope is more expensive and is less readily available in many places around the world.

Haluk et al. described using rigid ureteroscopy for ureter stent retrieval as an alternative that can be less expensive than the flexible cystoscope and may be less painful than the rigid cystoscope [4]. We have been routinely using both the rigid ureteroscope and the flexible cystoscope for the stent retrieval in our center. However, to our knowledge, there has never been a study comparing the clinical data for these two surgical modalities.

Previous studies have shown that using a flexible cystoscope to remove ureteral stent offer the advantage of being less painful in the male patient [14]. In the present study, we found that the perioperative and postoperative VAS for patients whose ureteral stent were removed using ureteroscopy were similar to those removed using flexible cystoscopy for both genders. In our opinion, because of its small caliber, rigid ureteroscopy can be an acceptable alternative for ureteral stent retrieval with less discomfort.

Irritable bladder symptoms and hematuria are the most common problems following flexible cystoscopy [15]. Donoghue et al. [16] and Kortman et al. [17] reported that 37% and 35.3% of men had pain over the bladder after flexible cystoscopy, respectively. 5% of the patients still had dysuria at 48 h postoperatively [15]. Dysuria is usually associated with trauma to the mucosa. Both rigid ureteroscopy and flexible cystoscopy tend to cause less trauma to the urethra than the rigid cystoscope. In our study, 3.4% of Group A and 3.2% of Group B experienced dysuria at postoperative day two, *P* > 0.05. There was no statistically significant difference between the two groups. Most of the dysuria resolved after 48 h without treatment.

Hematuria is the most common cause for hospital admission after stent removal [15]. No one in our study required hospitalization due to hematuria. Due to strong aversion to macroscopic hematuria in the Chinese culture, we routinely treat patients complaining of macroscopic hematuria with oral hemostatics for 3 to 5 days, a more aggressive therapy than in other countries. We found the incidence of hematuria and other postoperative complications were similar for both groups.

Flexible cystoscopes are more expensive and less durable than rigid cystoscopes. In addition, the per-use cost including the sterilization is higher than the per-use cost for the rigid scope. The deflection tip and outer bending rubber are the most common sites for damage. In a retrospective study by McGill et al., the mean failure time for flexible cystoscope was 134.6 procedures [18]. In this study, the per-use cost of the instrument was much higher in flexible cystoscopic group (RMB 723.1 or USD 107.9) than for the ureteroscope group (RMB 214.3 or USD 28.2). Two flexible cystoscopes were damaged during the 162 procedures. The flexible foreign body, forceps, was also more prone to breakage. In an in-vitro study, the maximum extraction force for the flexible graspers was only 1.3 kg [19]. Two flexible forceps were damaged during this study. The mean usage was 56.6 times. Moreover, a stone basket was occasionally required due to an inaccessible angle; this further escalated the cost. By contrast, no rigid ureteroscopes and only one pair of ureteroscopic grasping forceps were damaged after the 138 procedures.

The ureteroscopic method can be a reasonable alternative for the ureteral stent retrieval. The rigid ureteroscope, with its relatively low cost, is generally available in most of the hospitals and most of the urologists are proficient with its use. In fact, ureteroscopic extractions of stones have been widely performed even in lower income countries during the last two decades [20, 21].

The main limitation of this study is that it was a single-center study with a relatively small sample size. There would be unavoidable inherent bias. A multi-center prospective randomized controlled study with a larger sample size would be more ideal.

## Conclusion

Ureteral stent retrieval using a ureteroscope under topical anesthesia is as safe and effective as using a flexible cystoscope but at a lower cost. Rigid ureteroscopes may also be more available than flexible cystoscopes.

## Abbreviations

KUB: Kidneys, ureters, and bladder x-ray
RMB: Ren Min Bi
USD: United States dollar
VAS: Visual analogue pain scale
